# Extracellular Vesicles Alter Trophoblast Function in Pregnancies Complicated by COVID‐19

**DOI:** 10.1002/jev2.70051

**Published:** 2025-04-10

**Authors:** Thea N. Golden, Sneha Mani, Rebecca L. Linn, Rita Leite, Natalie A. Trigg, Annette Wilson, Lauren Anton, Monica Mainigi, Colin C. Conine, Brett A. Kaufman, Jerome F. Strauss, Samuel Parry, Rebecca A. Simmons

**Affiliations:** ^1^ Department of Obstetrics and Gynecology Perelman School of Medicine at the University of Pennsylvania Philadelphia Pennsylvania USA; ^2^ Center for Women's Health and Reproductive Medicine University of Pennsylvania Philadelphia Pennsylvania USA; ^3^ Center for Excellence in Environmental Toxicology University of Pennsylvania Philadelphia Pennsylvania USA; ^4^ Department of Pathology and Laboratory Medicine Children's Hospital of Philadelphia Philadelphia Pennsylvania USA; ^5^ Epigenetics Institute Perelman School of Medicine at the University of Pennsylvania Philadelphia Pennsylvania USA; ^6^ Department of Medicine University of Pittsburgh Pittsburgh Pennsylvania USA; ^7^ Institute for Regenerative Medicine Perelman School of Medicine at the University of Pennsylvania Philadelphia Pennsylvania USA; ^8^ Department of Genetics Perelman School of Medicine at the University of Pennsylvania Philadelphia Pennsylvania USA; ^9^ Department of Pediatrics Perelman School of Medicine at the University of Pennsylvania Philadelphia Pennsylvania USA; ^10^ Division of Neonatology Children's Hospital of Philadelphia Philadelphia Pennsylvania USA

**Keywords:** COVID‐19, extracellular vesicle cargo, extracellular vesicle origin, placental dysfunction, pregnancy

## Abstract

Severe acute respiratory syndrome coronavirus 2 (SARS‐CoV‐2) infection and resulting coronavirus disease (COVID‐19) cause placental dysfunction, which increases the risk of adverse pregnancy outcomes. While abnormal placental pathology resulting from COVID‐19 is common, direct infection of the placenta is rare. This suggests that pathophysiology associated with maternal COVID‐19, rather than direct placental infection, is responsible for placental dysfunction. We hypothesized that maternal circulating extracellular vesicles (EVs), altered by COVID‐19 during pregnancy, contribute to placental dysfunction. To examine this hypothesis, we characterized circulating EVs from pregnancies complicated by COVID‐19 and tested their effects on trophoblast cell physiology in vitro. Trophoblast exposure to EVs isolated from patients with an active infection (AI), but not controls, altered key trophoblast functions including hormone production and invasion. Thus, circulating EVs from participants with an AI, both symptomatic and asymptomatic cases, can disrupt vital trophoblast functions. EV cargo differed between participants with COVID‐19, depending on the gestational timing of infection, and Controls, which may contribute to the disruption of the placental transcriptome and morphology. Our findings show that COVID‐19 can have effects throughout pregnancy on circulating EVs, and circulating EVs are likely to participate in placental dysfunction induced by COVID‐19.

## Graphical abstract

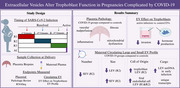

## 1 Introduction

Maternal severe acute respiratory syndrome coronavirus 2 (SARS‐CoV‐2) infection and resulting coronavirus disease (COVID‐19) are associated with an increased risk of pregnancy complications including preterm birth, hypertensive disorders of pregnancy, foetal growth restriction and pregnancy loss (Conde‐Agudelo and Romero 2022; Smith et al. 2023). Placental dysfunction is known to contribute to these complications, and placental pathology, including vasculopathies and inflammation, is frequently reported following an acute or even resolved infection during pregnancy (Corbetta‐Rastelli et al. 2023; Cribiu et al. 2021; Joshi et al. 2022; Patberg et al. 2021). This suggests that COVID‐19 has a long‐lasting effect on pregnancy by altering placenta function. Despite extensive reports of placenta morphological abnormalities following COVID‐19, little is known about the underlying mechanisms contributing to placental dysfunction and the related subsequent pregnancy complications. Analysis of the transcriptome in placentas that were exposed at different times during pregnancy can yield additional insight into potential mechanisms. Direct infection of the placenta is rare, which suggests that placental dysfunction is caused by the maternal response to SARS‐CoV‐2 infection (Patberg et al. 2021; Edlow et al. 2020; Mulvey et al. 2020).

Circulating extracellular vesicles (EVs) are altered by SARS‐CoV‐2 infection and contribute to COVID‐19‐induced organ damage (Balbi et al. 2021; Forte et al. 2023; George et al. 2023; Rosell et al. 2021; Traby et al. 2022). EVs are a means of cell‐to‐cell communication resulting from their ability to carry and transfer bioactive cargo that elicits signalling events in recipient cells. Compared to uninfected individuals, EV cargo composition in patients with COVID‐19 is significantly different, eliciting downstream systemic effects such as coagulopathy (Barberis et al. 2021; Fujita et al. 2021; Mao et al. 2021; Puhm et al. 2022) and inflammation (Forte et al. 2023; George et al. 2023; Barberis et al. 2021; Yim et al. 2022). For example, tissue factor protein abundance in EVs is increased in COVID‐19 and correlates with inflammation and disease severity demonstrating the influence that circulating EVs have on the systemic response to COVID‐19, thereby leading to organ dysfunction (Balbi et al. 2021; Rosell et al. 2021; Traby et al. 2022).

During normal pregnancy, the placenta releases EVs into the maternal circulation (Salomon et al. 2014; Sarker et al. 2014). Placental‐derived EVs promote maternal adaptation to support a healthy pregnancy including a shift in the maternal immune system to a tolerant state and the promotion of angiogenesis (Ratajczak et al. 2013; Bai et al. 2021). Placental‐derived EVs also affect trophoblast function through autocrine and paracrine signalling. Trophoblasts are a specialized cell type of the placenta that are responsible for invasion into maternal tissue to anchor the placenta, vascular remodelling for adequate placental blood flow, nutrient transport and hormone production for maternal and foetal signalling. EV signalling impairs normal trophoblast function, which is thought to contribute to the placental dysfunction underlying pregnancy complications (Ratajczak et al. 2013; Chang et al. 2018; Hromadnikova et al. 2019; Pillay et al. 2016; Salomon et al. 2016).

Therefore, we hypothesized that COVID‐19 alters circulating EV cargo, which has a functional consequence in the placenta.

## 2 Materials and Methods

### 2.1 Patient Cohort

The COMET study was conducted at the Hospital of the University of Pennsylvania (HUP) with Institutional Review Board approval (IRB#843277). Study participants received a description of the study and signed an informed consent before enrolment. Participants were enroled at the time of delivery in the COMET study between April 2020 and June 2022. Participants were tested for a SARS‐CoV‐2 infection by nasopharyngeal polymerase chain reaction (PCR) upon admission to the labour and delivery unit at HUP. Participants who tested positive at the time of delivery were enroled in the active infection (AI) group. Those participants who tested negative and had no known SARS‐CoV‐2 infection during their pregnancy were defined as Controls. Due to variation in humoral response and the duration of detectable antibodies, we chose not to define the Control population by a negative antibody test. However, there is the possibility a Control subject had an unknown history of SARS‐CoV‐2 infection. Participants with a negative test at delivery and a history of a documented positive SARS‐CoV‐2 PCR test during their pregnancy and greater than 14 days before enrolment were defined as having a resolved infection (R). The gestational age of SARS‐CoV‐2 infection was calculated, and participants were further divided into the trimester of infection (resolved infection in the first trimester (R1), resolved infection in the second trimester (R2) and resolved infection in the third trimester (R3)). All COVID‐19 cases were unvaccinated against SARS‐CoV‐2.

### 2.2 Clinical and Demographic Data Collection

Clinical characteristics, such as maternal age, self‐identified race, gestational age at infection and pregnancy outcomes, were extracted from the medical record (Table 1). The severity of COVID‐19 disease was determined based on the medical records and self‐reported symptoms and categorized based on the National Institute of Health and Society for Maternal‐Fetal Medicine definitions. Asymptomatic infection was defined as participants who tested positive but experienced no symptoms. Participants with a resolved symptomatic infection experienced a range of mild (cough, headache and fever) and/or moderate (shortness of breath) symptoms. Several participants with a resolved infection report hospitalization for COVID‐19; however, we do not have access to the clinical reasons for hospitalization and severity of disease. Clinical information for participants who tested positive for SARS‐CoV‐2 at the time of delivery (AI) is comprehensive and all subjects in this group either had an asymptomatic or mild case (Table 1).

**TABLE 1:** Subject demographics and placenta pathology.

Demographics	Controls (*n* = 32)	R1 (*n* = 14)	R2 (*n* = 20)	R3 (*n* = 21)	AI (*n* = 21)
Maternal age (years)	21–42 (Mean: 31.8)	25–43 (Mean: 31.6)	18 – 39 (Mean: 29.4)	21–42 (Mean: 30.3)	20–40 (Mean: 29.4)
Race: White	50%	42.9%	34%	28.6%	9.5%^*^
Race: Black	41%	50.0%	55%	61.9%^*^	76.2%^*^
Race: Asian	9.4%	0%	10%	0%	9.5%
Race: Other/Unknown	0%	7.1%	0%	9.5%	4.8%
GA at delivery (weeks)	37.3–41.1 (Mean: 39.3)	35.1–40.3 (Mean: 39.0)	33.7–41.3 (Mean: 38.4)	32.3–39.7 (Mean: 37.6)^*^	31–41 (Mean: 38.1)^*^
Mode of delivery (percentage vaginal)	75.0%	57.1%	70.0%	38.1%	57.1%
Parity (percentage 1st pregnancy)	21.9%	42.9%	50.0%	4.8%^*^	23.8%
Infant sex (percentage female)	56.3%	57.1%	35.0%	47.6%	47.6%

COVID severity	Controls (*n* = 32)	R1 (*n* = 14)	R2 (*n* = 20)	R3 (*n* = 21)	AI (*n* = 21)
Symptomatic	N/A	71.4%	85%	81.0%	14.3%^+^
Asymptomatic	N/A	28.6%	15%	19.0%	85.7%^+^
Hospitalized	N/A	14.3%	0%	23.8%	4.8%

Placenta pathology	Control (*n* = 26)	R1 (*n* = 9)	R2 (*n* = 17)	R3 (*n* = 19)	AI (*n* = 19)
MVM or FVM	26.9%	100%^*^	71%^*^	63%^*^	82.6%^*^
MVM	15%	44%^*^	47%^*^	37%	53%^*^
High grade MVM	0%	11%	18%^*^	16%^*^	11%
FVM	15%	78%^*^	59%^*^	42%^*^	47%^*^
>10% perivillous fibrin deposition	0%	11%	18%^*^	21%^*^	5%

*Note*: Participants enroled in the COMET study formed five groups (controls, resolved infection in the 1st trimester (R1), 2nd trimester (R2) and 3rd trimester (R3) and active infection (AI)). Maternal demographics including maternal age, race and gestational age (GA) at birth are reported. The severity of COVID‐19 during their pregnancy and placental pathology (maternal vascular malperfusion (MVM), foetal vascular malperfusion (FVM) and perivillous fibrin deposition) are reported.

* *p* < 0.05 chi‐squared test compared to Controls.

^+^ *p* < 0.05 compared to other COVID‐19 groups.

### 2.3 Sample Collection

Placentas were collected at the time of delivery and stored at 4°C until biopsy within 24 h (average 5.5 h, range 0.02–17.4 h). Four full‐thickness placental biopsies were collected from areas devoid of obvious pathology located equidistant between the placental cord insertion and the edge of the placenta. All placentas were examined by the pathology department at the HUP. Placentas were assessed using a systematic protocol that includes recording the trimmed placental weight, membrane insertion site, gross appearance, dimensions of the placental disc and umbilical cord insertion, length and diameter. Tissue was fixed in 10% formalin for histological assessment. A placenta pathologist reviewed all samples, and a subset of samples was also reviewed by additional placenta pathologists to ensure accuracy and reproducibility. Macroscopic and microscopic lesions were identified and classified according to the Amsterdam Placental Workshop Group 2014 classification (Freedman et al. 2021; Khong et al. 2016; Redline et al. 2021). One of the full‐thickness placental biopsies was stored in Trizol for RNA isolation and bulk RNA sequencing.

Blood was collected from the maternal periphery at delivery in an EDTA tube and spun at 1000 G for 10 min at room temperature to isolate plasma, which was aliquoted and stored at −80°C.

### 2.4 Placenta and BeWo RNA Isolation and Sequencing

Total RNA was isolated from placental biopsy samples using Qiagen RNEasy Plus Mini Kits (Cat# 74134 Qiagen, Hilden, Germany). Total RNA was isolated from syncytialized BeWo cells with the PicoPure RNA Isolation Kit (Cat# KIT0204 Applied Biosystems, Waltham, Massachusetts). Isolated RNA was sent to NovoGene for library preparation and sequencing.

RNA integrity and quantification were assessed using the RNA Nano 6000 Assay Kit of the Bioanalyzer 2100 System (Agilent Technologies, California, USA). RNA purity was determined using a NanoPhotometer spectrophotometer (IMPLEN, California, USA). A total of 1‐µg RNA per sample was used as input material for the RNA sample preparation. Sequencing libraries were generated using the NEBNext Ultra RNA Library Prep Kit for Illumina (NEB, USA) following manufacturer recommendations, and index codes were added to identify samples. Clustering of the index‐coded samples was performed on an Illumina Novaseq 6000 sequencer according to the manufacturer's instructions. After cluster generation, libraries were sequenced, and pair‐end reads were generated. Raw data (raw reads) of FASTQ format were processed through fastp, and clean data were obtained by removing reads containing adaptor and poly‐N sequences and reads with low quality. Pair‐end clean reads were aligned to the GRCh38/hg38 reference genome using Spliced Transcripts Alignment to a Reference (STAR) software. FeatureCounts were used to count the read number mapped to each gene. Then RPKM of each gene was calculated based on the length of the gene and the read count mapped to the gene. Differential gene expression between COVID‐19 groups and Controls was assessed by DESeq2. Differentially expressed genes were determined based on their adjusted *p* value (<0.05) and >1.5‐fold change. Functional analysis was conducted using Qiagen's ingenuity pathway analysis (IPA). The clusterProfiler R package was used to perform a Gene Ontology (GO) enrichment analysis of genes that were differentially expressed. Canonical pathways, transcriptional regulators and GO terms were considered significant if the *p* value was less than 0.05.

### 2.5 EV Isolation and Characterization

Serial centrifugation was utilized to isolate EVs from plasma. One millilitre of plasma was spun in an Eppendorf 5424 benchtop centrifuge at 2000 × *g* for 10 min at 4°C. The supernatant was then spun at 20,000 × *g* for 30 min at 4°C. The pellet was washed in 1‐mL filtered PBS and spun again at 20,000 × *g* for 30 min at 4°C. The pellet was re‐suspended in 100‐µL filtered PBS and EVs contained in this pellet were henceforth referred to as large EVs. The supernatant was spun by the Beckman Ultracentrifuge Optima Max TL using the TLA 120.2 rotor at 100,000 × *g* (48,000 RPM) for 90 min at 4°C. The pellet was washed with 1‐mL filtered PBS and spun again at 100,000 × *g* (48,000 RPM) for 90 min at 4°C. The small EV pellet was re‐suspended in 100‐µL filtered PBS.

EV isolation was confirmed by transmission electron microscopy, nanoparticle tracking and protein measurement as recommended by the MISEV guidelines (Théry et al. 2018). Transmission electron microscopy images were generated and resulting images were reviewed for the presence of EVs. EVs were analysed by Particle Metrix Zetaview nanoparticle tracking. Eleven fields were captured using the following parameters: sensitivity 80, frame rate 30, shutter 100, minimum brightness 1000, minimum area 10 and trace length 15. Representative histograms and TEM images for large and small EVs are included in Figure 2. CD9 protein abundance was determined by gel electrophoresis. Total protein was measured with a Qubit Protein Assay Kit, and EV suspension was evaporated by vacuum and re‐suspended in an electrophoresis buffer. Three large EV (5 µg) and small EV (20 µg) samples were loaded into BioRad Mini‐Protean TGX Gel 4%–20% polyacrylamide gels with Licor Chameleon Duo ladders (928–60,000) and run at 20 mA for 2 h. Proteins were transferred to nitrocellulose membrane via 200 mA over 3 h on ice. The membrane was blocked in Licor Intercept Blocking Buffer for 1 h at room temperature and then incubated with CD9 antibody (HI9a Biolegend Cat 312112) at 1:5000 overnight at room temperature. The membrane was then incubated with Licor IRDye 800CW streptavidin (926‐32230) at 1:5000 for 2 h at room temperature, and the membrane was imaged by Licor Odyssey.

### 2.6 Flow Cytometry on EVs

EVs surface protein expression was determined by flow cytometry following the MISEV guidelines (Théry et al. 2018). EVs were re‐suspended at 1 × 10^8^/10 µL of filtered PBS. Di‐8‐ANEPPS (Invitrogen Cat# D3167) was re‐constituted in ethanol as per manufacturer instruction and further diluted to 1:1000 in filtered PBS. Antibodies were spun at 20,000 × *g* for 30 min at 4°C immediately before use. Ten microlitres of EV suspension was incubated in ANEPPS (10 µL) and antibodies, 1.25‐µL CD45‐Ry586 (Cat# BD568135), 1.25‐µL CD41a‐ PE/Cy7 (Cat# BDB561424), 1.25‐µL PLAP‐ eFlour660 (Fisher Cat# 50‐112‐4573) and 1.25‐µL CD34‐ PE/CF594 (Cat# BDB562449) and 3‐µL CD31‐AF700 (Biolegend Cat# 50‐207‐2950), for 30 min at room temperature. Four hundred and seventy microlitres of filtered PBS was added before samples were measured by BD Symphony A1 cytometer, which has improved sensitivity for small particles. Negative controls included the following: antibodies alone, EVs without Di‐8‐ANEPPS and EVs treated with 1% triton. Data were analysed using FlowJo software. EVs were identified by Small Particle Side Scatter (SP‐SSC) and expression of Di‐8‐ANEPPS and the relative proportion that expresses cell‐specific surface proteins was determined by antibody detection.

### 2.7 In Vitro Trophoblast EV Co‐Culture

Human tissue was collected from the Penn Family Planning and Pregnancy Loss Center under an IRB approved by the University of Pennsylvania (#827072). Tissue was collected from elective (not performed for genetic or congenital abnormalities) terminations from patients with no pre‐existing medical conditions or any pregnancy complications. First‐trimester placental tissue (gestational ages 6 weeks 0 day, 8 weeks 1 day and 10 weeks 6 days) was used because the invasiveness of trophoblasts is higher in early compared to later in pregnancy. Extravillous trophoblasts (EVTs) were isolated from fresh first‐trimester placenta based on an EVT outgrowth–based protocol established by Gram et al. (Anton et al. 2012; Anton et al. 2019; Getsios et al. 1998; Graham et al. 1992; Park et al. 2022). In brief, villous tissue was finely minced and cultured at 37°C and 5% CO_2_ in RPMI 1640 media with 20% FBS. After attachment, EVT outgrowth occurs, and those cells were isolated. Isolated EVTs were confirmed by staining for HLA‐G and CK7.

EVT invasion was measured using the MilliporeSigma Chemicon QCM Collagen Cell Invasion Assay (Cat# ECM558). EVTs were added to the trans‐well invasion plate with EV‐depleted media and large and small EVs each at 1 × 10^6^/mL. Cells were incubated at 37°C and 5% CO_2_ for 48 h. Cells that invaded through the collagen membrane were quantified using a fluorescent plate reader (SpectraMax).

BeWo cells, subclone B30, were cultured in 75‐cm^2^ flasks (Fisher Scientific) at 37°C and 5% CO_2_ in media (DMEM/F12, 10% FBS, 1% P/S, 1% L‐alanyl‐L‐glutamine). EV‐depleted media were made with EV‐depleted FBS (Gibco A2720801) and used for cell culture experiments. Cells were plated at 250,000 cells/well in a six‐well plate and 1.5 mL of EV‐depleted media was added. Cells adhered for 24 h before adding 1‐µg/µL forskolin, a cAMP producer, to promote syncytialization for an additional 24 h. Large and small EVs were re‐suspended in EV‐depleted media at 1 × 10^6^/mL and added to BeWo cells for an additional 24 h. At the time of harvest, cell media were collected, and cells released with 0.25% trypsin. Cells were washed and collected as pellets for total DNA measurement and RNA isolation and sequencing. Cell media were spun at 500 × *g* to clear cell debris, and the supernatant was stored for future hormone measurement. Hormones were measured by Penn Fertility Care using Elecsys HCG+β (Cat# 03271749, Roche Diagnostics) and Elecsys Progesterone III (Cat# 07092539, Roche Diagnostics).

### 2.8 EV mtDNA Measurement

mtDNA was isolated and quantified from large and small EVs by TaqMan‐based quantitative polymerase chain reaction (qPCR). We measured mtDNA in a range of large and small EV concentrations (1 × 10^7^ to 6 × 10^9^) and determined that 2 × 10^7^ large EVs and 3 × 10^9^ small EVs were necessary to reliably and robustly measure mtDNA. As previously described, we quantified mitochondrial‐encoded human NADH: ubiquinone oxidoreductase core subunit 1 (ND1) as previously described (Ware et al. 2020). The qPCR reactions were performed in triplicates using a QuantStudio 5 Real‐time PCR System (Thermo Fisher) using the following thermocycling conditions: 95 °C for 20 s followed by 40 cycles of 95 °C for 1 s, 63 °C for 20 s and 60 °C for 20 s. Serial dilutions of pooled human placenta DNA quantified for copies of ND1 (copies per microlitre) by digital PCR (dPCR) were used as a standard curve. The mtDNA amount per EV was determined by normalizing the resulting abundance by the number of EVs in the starting material. We calculated the Pearson correlation coefficient to determine the strength of the relationship between gestational age at infection and the abundance of EV mtDNA.

### 2.9 EV mRNA Sequencing

Total RNA was isolated from large and small EVs isolated from 500 µL of plasma. Isolated EVs were treated with RNAseA (0.02 µg/µL) (Invitrogen Cat# 12091021) for 20 min at 37°C to degrade extravesicular RNA. Enzyme activity was stopped by freezing samples at −80°C for 5 min and immediate re‐suspension in Trizol. Nucleic acids were isolated via BCP co‐incubation, precipitated by isopropanol and washed in ethanol. mRNA libraries were prepared from total RNA using the SMART‐Seq protocol (Trombetta et al. 2014). Briefly, RNA was reverse transcribed using Superscript II (Invitrogen, Cat#18064014). The cDNA was amplified with 20 cycles and cleaned up with AMPure XP beads (Beckman Coulter Cat#A63881). cDNA was quantified with Qubit dsDNA HS Assay Kit (Life Technologies, Inc. Cat#Q32851), and 2 ng of each sample was used to construct a pool of uniquely indexed samples (Illumina Cat# FC‐131‐1096). A second amplification was performed with 12 cycles and cleaned up with AMPure XP beads. The final library was sequenced on a NextSeq 1000. Data were mapped against the hg19 genome using RSEM and normalized to transcripts per million (tpm) (Yukselen et al. 2020).

To determine unique expression, we filtered genes to those that had greater than 5 tpm. Unique genes had no expression (≤5 tpm) in all samples in the reference group and had expression (>5 tpm) in the majority (≥ 50% of the samples) in the comparison group (data in Table 2). Expression of unique genes in Controls, but not COVID‐19 groups are shown in Figure 6. These strict criteria identified genes in each group that were uniquely expressed, and those genes were considered for subsequent analysis.

**TABLE 2:** Unique genes identified in EVs isolated from COVID‐19 cases.

Uniquely expressed transcripts in COVID EVs					
Gene symbol	Gene name	Group(s) with unique expression	Cellular function	High trophoblast expression	Increased in pregnancy
Large EVs					
*YY1AP1*	YY1‐Associated Protein 1	R1, R2	Transcription		
*MOSPD1*	Motile Sperm Domain Containing 1	R1, AI	Transcription		
*RYBP*	RING1 and YY1 Binding Protein	R1, AI	Transcription	Y	
*H1‐4*	H1.4 Linker Histone, Cluster Member	R2, R3	Unknown		
*A2M*	Alpha‐2‐Macroglobulin	R1	Immune		
*KIFAP3*	Kinesin‐Associated Protein 3	R1	Chromosome movement		Y (Rehman et al. 2003)
*MXD1*	MAX Dimerization Protein 1	R1	Proliferation		
*PEX19*	Peroxisomal Biogenesis Factor 19	R1	Oxidative stress	Y	
*TOB1*	Transducer of ERBB2, 1	R1	Proliferation		
*HK1*	Hexokinase 1	R2	Metabolism	Y	
*LY9*	Lymphocyte Antigen 9	R2	Immune		Y (Whittington et al. 2018)
*POLR3C*	RNA Polymerase III Subunit C	R2	Nucleic acid binding activity		
*SPNS3*	SPNS Lysolipid Transporter 3, Sphingosine‐1‐Phosphate (Putative)	R2	Transporter activity		
*WDR46*	WD Repeat Domain 46	R2	Nucleic acid binding activity		
*CDC34*	Cell Division Cycle 34, Ubiquitin Conjugating Enzyme	R3	Ubiquitination		
*FUNDC2*	FUN14 Domain Containing 2	R3	Metabolism		
*LINC‐PINT*	Long Intergenic Non‐Protein Coding RNA, P53‐Induced Transcript	R3	Unknown		
*R3HCC1*	R3H Domain and Coiled‐Coil Containing 1	R3	Nucleic acid binding activity		
*ATPSCKMT*	ATP Synthase C Subunit Lysine N‐Methyltransferase	AI	Mitochondrial ATP synthesis		
*DTWD1*	DTW Domain Containing 1	AI	Translation		
*FBXL4*	F‐Box and Leucine‐Rich Repeat Protein 4	AI	Ubiquitination		
*FRA10AC1*	FRA10A‐Associated CGG Repeat 1	AI	Transcription		
*FOXP1*	Forkhead Box P1	AI	Transcription		
*GOLGA4*	Golgi A4	AI	Protein and lipid transport	Y	Y (Rehman et al. 2003)
*GPBP1L1*	GC‐Rich Promoter Binding Protein 1 Like 1	AI	Transcription		
*IL1R2*	Interleukin 1 Receptor Type 2	AI	Immune		
*KIAA1143*	KIAA1143	AI	Unknown		
*LINC01410*	Long Intergenic Non‐Protein Coding RNA 1410	AI	Unknown		
*NEAT1*	Nuclear Paraspeckle Assembly Transcript 1	AI	Transcription	Y	
*WDR26*	WD Repeat Domain 26	AI	Cell‐cycle progression and gene regulation	Y	
*XRN1*	5'‐3' Exoribonuclease 1	AI	mRNA degradation		Y (Peng et al. 2018)
*ZNF638*	Zinc Finger Protein 638	AI	Transcription		
Small EVs					
*MYL4*	Myosin Light Chain 4	R1, AI	Motor protein		Y (Maron et al. 2007)
*C18orf32*	Chromosome 18 Open Reading Frame 32	R1, AI	Immune	Y	
*CAPG*	Capping Actin Protein, Gelsolin Like	R2, AI	Motor protein		
*CTSS*	Cathepsin S	R3, AI	Immune		Y (Song et al. 2007)
*CYREN*	Cell Cycle Regulator of NHEJ	R1	DNA repair		
*CCDC124*	Coiled‐Coil Domain Containing 124	R1	Transcription		
*CD27‐AS1*	CD27 Antisense RNA 1	R1	Immune		
*GNB2*	G Protein Subunit Beta 2	R1	G protein signalling		
*PHF5A*	PHD Finger Protein 5A	R1	Immune	Y	
*RBM8A*	RNA Binding Motif Protein 8A	R1	Transcription	Y	
*SIGMAR1*	Sigma Non‐Opioid Intracellular Receptor 1	R1	Calcium signalling	Y	
*CSF3R*	Colony Stimulating Factor 3 Receptor	R2	Immune	Y	
*ESD*	Esterase D	R2	Metabolism		
*GTF2IRD2*	GTF2I Repeat Domain Containing 2	R2	Transcription		
*SLC1A5*	Solute Carrier Family 1 Member 5	R2	Metabolism	Y	Y (Chen et al. 2006)
*AUP1*	AUP1 Lipid Droplet Regulating VLDL Assembly Factor	R3	Ubiquitination	Y	
*MFF*	Mitochondrial Fission Factor	R3	Mitochondrial and peroxisomal fission		
*ARCN1*	Archain 1	AI	Vesicle	Y	
*CKAP2*	Cytoskeleton‐Associated Protein 2	AI	Proliferation		Y (Fulghum et al. 2022)
*CSNK2B*	Casein Kinase 2 Beta	AI	Metabolism		
*CYP27C1*	Cytochrome P450 Family 27 Subfamily C Member 1	AI	Metabolism		
*GRHL1*	Grainyhead‐Like Transcription Factor 1	AI	Epithelial development	Y	
*HSP90B1*	Heat Shock Protein 90 Beta Family Member 1	AI	Molecular chaperone	Y	Y (Prater et al. 2021)
*KIF5B*	Kinesin Family Member 5B	AI	Protein‐binding activity		
*LINC01123*	Long Intergenic Non‐Protein Coding RNA 1123	AI	Unknown		
*LOC728323*	Unknown	AI	Unknown		
*MANBAL*	Mannosidase Beta Like	AI	Membrane protein		Y (Rabaglino et al. 2023)
*MUC22*	Mucin 22	AI	Membrane protein		
*MYLK*	Myosin Light Chain Kinase	AI	Contractile activity		
*OR4F17*	Olfactory Receptor Family 4 Subfamily F Member 17	AI	Vesicle		
*PARP9*	Poly (ADP‐Ribose) Polymerase Family Member 9	AI	Immune		
*RNF2*	Ring Finger Protein 2	AI	Transcription		
*SAR1A*	Secretion‐Associated Ras Related GTPase 1A	AI	Vesicle	Y	
*SHC4*	SHC Adaptor Protein 4	AI	Proliferation		
*SRSF8*	Serine‐ and Arginine‐Rich Splicing Factor 8	AI	Transcription	Y	
*TMCC2*	Transmembrane and Coiled‐Coil Domain Family 2	AI	Metabolism	Y	
*TRIM4*	Tripartite Motif Containing 4	AI	Immune		
*TWF2*	Twinfilin Actin Binding Protein 2	AI	Actin and ATP binding site		
*VTA1*	Vesicle Trafficking 1	AI	Vesicle		
*ZNF484*	Zinc Finger Protein 484	AI	Transcription		

*Note*: Transcripts are carried by large EVs or small EVs isolated from COVID‐19 cases that are absent in Controls. The listed transcripts are not detected in EVs isolated from Controls but are present in the identified COVID‐19 group(s). The cellular function, expression in trophoblasts and pregnancy‐associated expression of each transcript are listed as well (reference listed).

### 2.10 Statistical Analysis

Statistical analysis was performed using GraphPad Prism. Differences in participant demographics and outcomes were tested by a chi‐squared test and considered statistically different if *p* < 0.05. Data were tested for normality and either parametric or non‐parametric tests were used to determine significance. Data points were identified as outliers and removed if they exceeded two times the standard deviation from the mean. A one‐way ANOVA tested for a difference within all groups and subsequent post hoc *t*‐tests or Kruskal–Wallis determined the significance of each COVID‐19 group compared to Controls. Pearson's correlation was used to determine a correlation between gestational age at infection and mtDNA abundance. A *p* value less than 0.05 was considered significant. Differential gene expression was determined to be significant if the adjusted *p* value was less than 0.05 and the fold change greater than 1.5.

## 3 RESULTS

### 3.1 Pregnancy Outcomes Following Active and Resolved SARS‐CoV‐2 Infection

Participants were enroled at the time of delivery between July 2020 and August 2022. Controls had no known SARS‐CoV‐2 infection during pregnancy. COVID‐19 cases were divided based on the gestational timing of infection and either had a resolved infection that occurred in the 1st, 2nd or 3rd trimester (R1, R2 and R3) or had an AI at the time of delivery (AI) (Table 1). All patients admitted to the labour and delivery unit underwent PCR testing for SARS‐CoV‐2 at admission. There were no significant differences in maternal age between groups, but there were significantly more Black participants with an infection in the third trimester (resolved or active) compared to non‐infected individuals (Controls) (R3 *p* = 0.046, AI *p* = 0.011). Participants with an infection in the third trimester, resolved or active, also had an earlier gestational age at delivery than Controls (R3 *p* < 0.001, AI *p* = 0.034) (Table 1). Not surprisingly, the AI group, which underwent universal screening for SARS‐CoV‐2 at admission for delivery, had a higher incidence of asymptomatic infection than those with resolved infection (*p* < 0.0001).

While our study was somewhat limited by relatively small numbers (*n* = 15–32) in each participant group, we found that the type of adverse outcome differed depending on the gestational timing of infection (Table S1).

### 3.2 Placental Pathology in COVID‐19

Abnormal placental pathology is commonly reported in patients with active and resolved SARS‐CoV‐2 infections (Corbetta‐Rastelli et al. 2023). However, there has yet to be a comprehensive assessment of placenta morphology following maternal infection at various gestational ages. Similar to previous reports (Corbetta‐Rastelli et al. 2023; Cribiu et al. 2021; Joshi et al. 2022; Patberg et al. 2021; Baergen et al. 2020; Sharps et al. 2020), we found maternal and foetal vascular malperfusion (MVM and FVM) lesions were increased among patients with pregnancies complicated by COVID‐19 (Table 1). Interestingly, high‐grade MVM and perivillous fibrin deposition were increased in participants with a resolved infection that occurred in the second or third trimester, but not the first trimester or with an AI.

### 3.3 Timing of COVID‐19 Impacts the Placental Transcriptome

**FIGURE 1:**
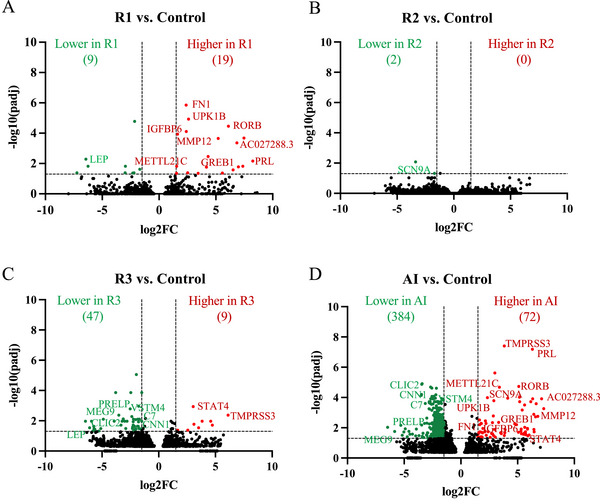
Differential gene expression in the placentas of patients with COVID‐19 during pregnancy is represented by volcano plots. (A–D) The number and direction of differentially expressed genes between Controls (*n* = 5) and resolved infections in the first trimester (R1) (A), second trimester (R2) (B) and third trimester (R3) (C), and active infection (AI) (D) is listed at the top of each graph. The gene name for those transcripts with differential expression in more than one COVID‐19 group compared to Control is listed. (*n* = 3–5/group).

To gain insight into potential novel pathways that might be affected by COVID‐19 during pregnancy, we performed bulk RNA‐seq on the placental transcriptome using biopsies from the placenta collected at delivery in COVID‐19 cases and controls. Not surprisingly, AI was associated with significant differences in gene expression in the placenta. There were 72 upregulated genes and 384 downregulated genes in AI compared to Control placentas (Figure 1 and Table S2). Gene expression was also altered in recovered infections, but the magnitude of change was smaller (Figure 1). It is worth noting that in each of the four groups, there was differential expression of genes that regulate mitochondria activity, which implies a common dysfunctional pathway resulting from COVID‐19.

### 3.4 Shared Changes to the Placental Transcriptome Regardless of Infection Timing

In addition, several other genes were differentially expressed in one or more COVID‐19 groups. There were several transcripts that were increased in placenta from R1 and AI groups compared to Controls. Several of these genes regulate fibrosis (*RORB*, *FN1*, *IGFB6*, *MMP12* and *AC027288.3*) (Ohmaru‐Nakanishi et al. 2018; Li et al. 2022; Foote et al. 2019; Fang et al. 2023; Liso et al. 2022) suggesting that this pathway plays an important role in placenta pathology in COVID‐19. Moreover, MMP12 regulates spiral artery remodelling and reduced MMP12 activity contributes to the development of preeclampsia (Chakraborty et al. 2016). Similarly, increased expression of *FN1* slice variants containing Extra Domain A promotes inflammation via Toll‐like receptor 4 (TLR4) activation (Mogami et al. 2013; Okamura et al. 2001) and is associated with an increased risk of preeclampsia (Ohmaru‐Nakanishi et al. 2018; Mogami et al. 2013; Okamura et al. 2001). Finally, *GREB1* was upregulated in R1 and AI placenta compared to Controls. GREB1 interacts with the progesterone (P4) receptor to regulate P4‐responsive genes (Camden et al. 2017; Chadchan et al. 2024) and GREB1 promotes maternal tissue remodelling (Camden et al. 2017).

Several of the downregulated genes in AI compared to Controls were also downregulated in the R3 placenta compared to Controls (*PRELP*, *MEG9*, *VSTM4*, *CLIC2*, *C7* and *CNN1*). Interestingly, *CNN1*, which encodes calponin, is expressed by smooth muscle cells and expression changes are associated with spiral artery remodelling (Biswas Shivhare et al. 2014; Whitley and Cartwright 2010). Further, SARS‐CoV‐2 infection is known to disrupt complement pathways (Afzali et al. 2022) and the gene‐encoding complement C7 was downregulated in R3 and AI placenta compared to Controls. The complement system plays a dual role in pregnancy in that it protects the placenta from pathogen infection and participates in spiral artery remodelling (Chighizola et al. 2020). *STAT4*, which encodes the signal transducer and activator of transcription 4, is a key activator of immune‐regulating genes and was upregulated in R3 and AI placentas compared to Controls. STAT4‐mediated pathways are disrupted in preeclampsia and circulating levels are elevated in patients with preeclampsia (Li et al. 2023; Zhang et al. 2020).

### 3.5 Pathways Altered in the Placenta by COVID‐19

Statistically significant disruptions in canonical pathways, as determined by IPA, include inflammation and fibrosis in the placentas from R1, R3 and AI compared to Controls (Table S3). This unbiased approach based on differential gene expression supported our placental histopathology findings of inflammation and fibrosis in these placentas. Thus, despite the long period of time following the resolution of maternal SARS‐CoV‐2 infection, genes that regulate fibrosis and inflammation were altered, and pathological evidence of fibrosis and inflammation were apparent in the placenta regardless of the timing of infection.

We also used IPA to predict transcriptional regulators of genes differentially expressed in the placenta from pregnancies complicated by COVID‐19. Interestingly, genes that encode for growth factors (IGF1, *IGF2*, *FGF3, FGF19* and *TGFB1*), immune‐regulating proteins (*JUN, TNF, IL1B, IL13, IL6, IL10, IL4* and *IFNG*) and hormone‐regulating proteins (*PRLH, LEPR, ESR1* and *PGR*) were the top predicted transcriptional regulators (Table S4).

### 3.6 Sustained Effects on Circulating EVs Following COVID‐19 in Early Pregnancy

**FIGURE 2:**
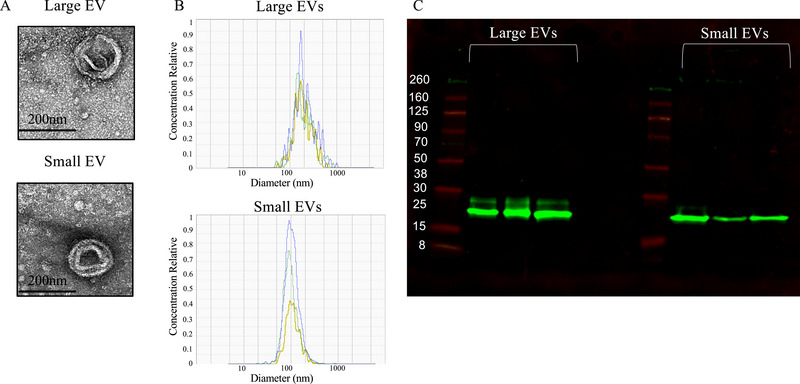
Extracellular vesicles isolated from the plasma by serial centrifugation. (A) Representative transmission electron microscope images demonstrated EV structure including a lipid membrane. (B) Nanotracking analysis was used to determine the size of particles in three Control samples. (C) CD9 expression evaluated by immune blotting was abundant in isolated particles in three Control samples.

As discussed above, SARS‐CoV‐2 rarely infects the placenta, implying a distal signal. We hypothesized that maternal circulating EVs play a role in mediating placental dysfunction associated with COVID‐19. Therefore, we characterized EVs isolated from maternal plasma collected at delivery to determine if COVID‐19 altered the EV profile. We isolated large and small EVs as they carry distinct cargo with distinct functional effects. We confirmed the presence of large and small EVs in isolated particles by electron microscopy (Figure 2A). Large and small EVs had the expected size distribution of EVs (Figure 2B). Large EV diameter was on average of 279.2 ± 26.49 nm and small EV diameter was on average 113.4 ± 14.07 nm. EVs smaller than 88 nm were not collected by our isolation method. Both large and small EVs abundantly expressed the EV‐related tetraspanin CD9 (Figure 2C).

**FIGURE 3:**
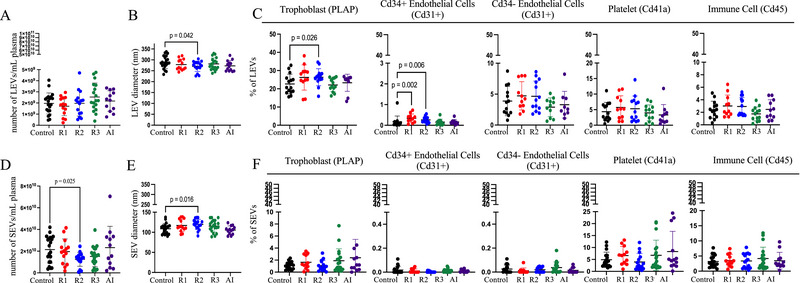
Circulating EVs were persistently altered in participants who experienced COVID‐19 in the second trimester. (A) The number of LEVs in the circulation at the time of delivery (*n* = 14–22/group). (B) The diameter of LEVs in the circulation (*n* = 14–22/group). (C) Relative frequency of LEVs derived from trophoblasts, endothelial cells, platelets and immune cells (*n* = 11–16). (D) The number of small EVs in circulation at the time of delivery (*n* = 14–22/group). (E) The diameter of small EVs in the circulation (*n* = 14–22/group). (F) The relative frequency of small EVs derived from endothelial cells, platelets, immune cells and trophoblasts (*n* = 13–20/group). All data are presented as mean ± SD. All analyses were performed by one‐way ANOVA or the Kruskal–Wallis test, followed by post hoc tests. Comparisons were made between Controls and resolved infection in the first trimester (R1), second trimester (R2), third trimester (R3) and active infection (AI).

EV characteristics, including concentration and size distribution, revealed long‐lasting alterations in patients with a resolved infection. The diameter of small EVs was significantly increased in resolved infections compared to Controls (108.7 vs. 117.2 nm, *p* = 0.023). However, the difference in concentration was not significant (2.15×10^8 ^vs. 1.56 × 10^8^ EVs/µL plasma, *p* = 0.074). When resolved infections were categorized by timing during gestation, we found that small EVs isolated from R2 participants had an increased diameter and reduced concentration, but small EVs isolated from R1 and R3 participants were not different from Controls (Figure 3D,E). The diameter of large EVs isolated from R2 patients was decreased but there was no change in their number (Figure 3A,B).

### 3.7 Altered Cell of Origin of EVs in Patients With COVID‐19 in Early Pregnancy

Characterizing the source of circulating EVs provides biological information about the tissue and cell type of origin and its functional state. We identified EVs, using flow cytometry, that originated from maternal endothelial cells (CD31+ CD34−), foetal endothelial cells (CD31+ CD34+), platelets (CD41a+), immune cells (CD45+) and trophoblasts (PLAP+) (Figure 3C,F).

Trophoblast‐derived PLAP+ EVs comprised the largest proportion of circulating large EVs (Figure 3C). The percentage of PLAP+ EVs was increased in the circulation of R2 compared to Controls suggesting the placenta secreted more large EVs into circulation. Interestingly, we found a subset of endothelial‐derived EVs that also express CD34, suggesting that these EVs originated from foetal endothelial cells (Parant et al. 2009). Foetal endothelial cell–derived large EVs (CD34+ CD31+) were also elevated in R1 and R2 compared to Controls. The percentage of small EVs from the placenta was not altered by COVID‐19 (Figure 3F). In fact, there was no difference in the percentage of small EVs from any cell type measured. This suggests that placenta‐derived large, but not small EVs, were altered by COVID‐19 in early pregnancy.

### 3.8 Circulating EVs From COVID‐19 Pregnant Patients Alter Trophoblast Function In Vitro

The placenta is made up of the following three main functional cell types: (1) syncytiotrophoblast cells, which are responsible for nutrient transport and hormone production; (2) cytotrophoblast cells, the replicating precursors of the syncytiotrophoblast and (3) EVTs, which invade deep into the uterus to anchor the placenta and enable blood and nutrient flow to the foetus. Therefore, we tested the capacity of circulating EVs isolated from participants with an AI or Controls to alter the function of trophoblast cell types. We focused our in vitro experiments on EVs isolated from participants with an AI compared to Controls because the changes in placental pathology and transcriptome were the greatest in AI cases compared to Controls.

**FIGURE 4:**
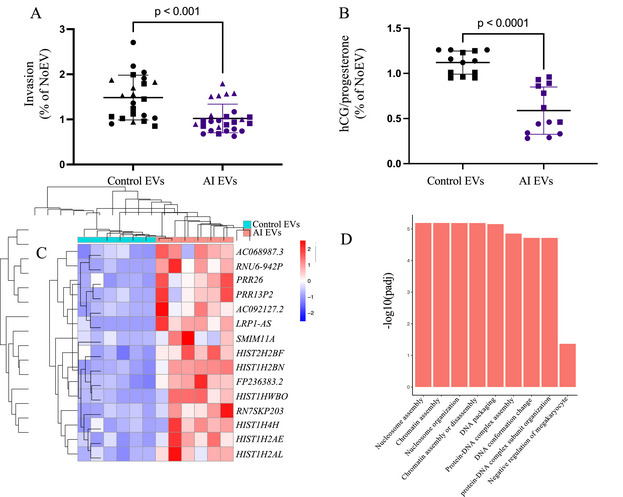
Trophoblast function was disrupted by exposure to EVs isolated from patients with an active infection (AI) compared to Controls. (A) Extravillous trophoblasts (EVTs) were isolated from three placentas (circle (gestational age 6 weeks 0 day), triangle (gestational age 8 weeks 1 day) and square (gestational age 10 weeks 6 days)) and exposed to Control or AI EVs (*n* = 9–10/group, three experiments). Invasion was calculated and normalized to the invasion of EVTs derived from the same placenta not exposed to EVs (noEVs). All data are presented as mean ± SD. (B) Human chorionic gonadotropin (hCG) and progesterone were measured in the media of forskolin‐treated (syncytialized) BeWo cells. The ratio of hCG to progesterone was normalized to hormone production by cells not exposed to EVs (noEVs). The results of two experiments (identified by symbol shape) are reported in (B). (*n* = 6–7/group, two experiments). All analyses were performed by one‐way ANOVA or Kruskal–Wallis test, followed by post hoc tests. (C) Following EV exposure, BeWo cell transcriptome was measured, and the top differentially expressed genes comparing the cellular response to AI EVs to Control EVs are listed in the heat map (red represents increased and blue represents decreased expression). (D) The biological processes altered by AI EVs compared to Control EVs were determined by Gene Ontology enrichment analysis.

The effect of AI EVs on EVT invasion was assessed using a collagen gel invasion assay. We found that EVT invasion was significantly reduced by exposure to AI EVs compared to Control EVs (31.2% reduction *p* < 0.001) (Figure 4A).

To study syncytiotrophoblasts, we used the BeWo choriocarinoma cell line that is commonly used to study this trophoblast lineage. After the addition of forskolin, BeWo cells syncytialize forming cells that mimic the syncytiotrophoblast, including the production of placental hormones (hCG and progesterone). Syncytiotrophoblast hormone production is essential for the maintenance of pregnancy. The ratio of hCG to progesterone in the media of syncytialized BeWo cells was significantly reduced following exposure to AI EVs compared to Control EVs (Figure 4B).

To identify novel pathways that may contribute to trophoblast dysfunction, we analysed the BeWo transcriptome following EV exposure. AI EVs significantly altered gene expression in BeWo cells compared to Control EVs. Multiple genes were dysregulated including genes that encode for long non‐coding RNA genes and histone proteins (Figure 4C). This suggests that DNA packaging and transcription are disrupted in trophoblasts exposed to AI EVs compared to Control EVs. Consistent with this, Biological Processes, determined by GO analysis, and top canonical pathways, identified by IPA, were related to cellular transcription and DNA repair (Figure 4D and Table S5). The pathways disrupted by AI EVs suggest a generalized effect on trophoblast gene expression that led to disrupted hormone production.

### 3.9 Increased mtDNA Content in LEVs Following COVID‐19 in Early Pregnancy

**FIGURE 5:**
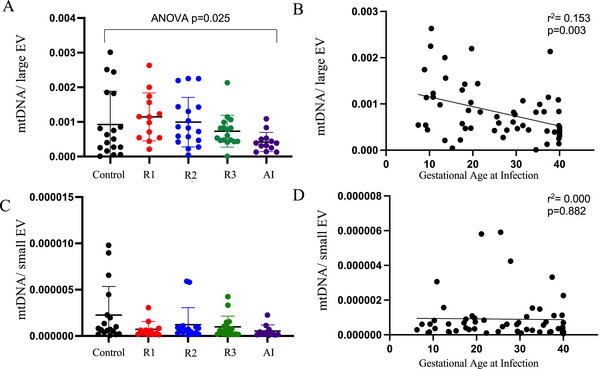
Large EV abundance of mtDNA is inversely correlated with gestational timing of infection. The amount of mtDNA in each large EV (A) and small EV (C) is reported for each group (*n* = 13–20/group). Data are presented as mean ± SD and tested by ANOVA followed by post hoc tests. Independent pairwise comparisons were made between Controls and resolved infection in the 1st trimester (R1), second trimester (R2), third trimester (R3) or active infection (AI). The Pearson correlation between gestational age at infection and mtDNA content is reported for large EV (B) and small EV (D).

Analysis of the placental transcriptome following COVID‐19 identified differentially expressed genes indicative of mitochondrial dysfunction (Tables S2 and S3). To determine if circulating EVs were enriched in mitochondrial cargo, we measured mitochondrial DNA (mtDNA) content (Figure 5A,C) and found that mtDNA was more abundant in large compared to small EVs. In contrast, nuclear DNA was not consistently measurable in all samples. Additionally, the abundance of mtDNA in large, but not small EVs, inversely correlated with the gestational timing of COVID‐19 (Figure 5B,D).

### 3.10 COVID‐19 During Pregnancy Alters EV RNA Cargo

EVs contain small and larger (mRNAs) and long noncoding RNAs, however, small RNAs are the most commonly studied EV cargo (Prieto‐Vila et al. 2021). mRNAs encapsulated within EVs are transferred to recipient cells and translated into proteins, altering the behaviour of the recipient cells (Deregibus et al. 2007; Ratajczak et al. 2006; Valadi et al. 2007; Wei et al. 2017). In addition, there are epigenetic regulators, such as microRNAs, DNA methylation and histone‐modifying enzymes, contained in EVs that can also influence the function of the recipient cell. Therefore, we profiled the mRNA content of circulating EVs to determine if there were differences dependent on the gestational timing of COVID‐19. We sequenced an average of 2946 gene‐associated transcripts in large EVs and 1947 in small EVs.

The most abundant mRNA transcripts in EVs were common to all groups. However, we also identified transcripts that were either uniquely expressed in COVID‐19 groups (i.e., absent in Controls) or uniquely expressed in Controls, and absent in one or more COVID‐19 groups. Multiple transcripts were uniquely expressed in large EVs from the COVID‐19 groups including *YY1AP1*, *MOSPDI, RYBP* and *H1‐4* (Table 2). The proteins encoded by these mRNAs are related to transcription, except for *HI‐4*, which has an unknown cellular function. In small EVs, *MYL4*, *C18orf32*, *CAPG* and *CTSS* transcripts were uniquely present in EVs isolated from COVID‐19 groups (Table 2). These transcripts encode a motor (*MYL4* (myosin light chain 4)) and immune (*C18orf32* (Putative NF‐Kappa‐B‐Activating Protein 200), *CAPG* (macrophage capping protein) and *CTSS* (cathepsin S)) proteins. This suggests that COVID‐19 alters immune‐related small EV cargo. Many other transcripts were uniquely detected in EVs isolated from individual COVID‐19 groups (Table 2). The unique transcriptome suggests that COVID‐19 increased expression of these genes making their transcripts more available for EV packaging or increased specific transcript loading into EVs.

**FIGURE 6:**
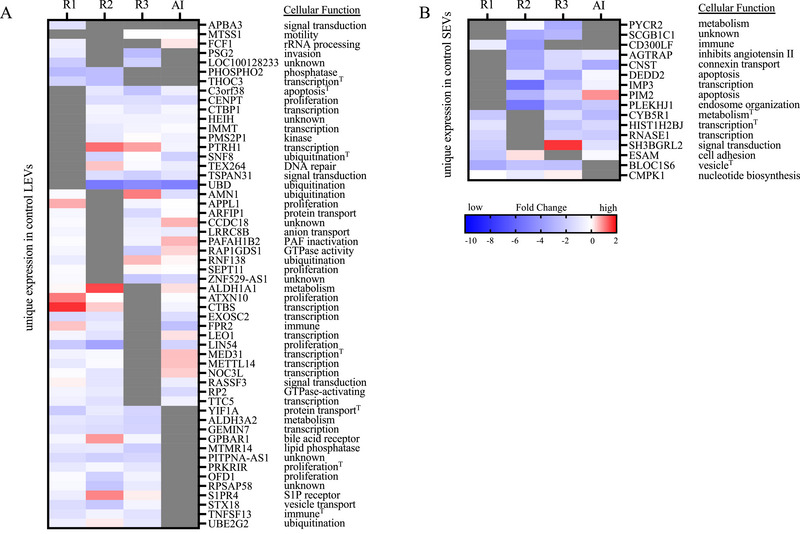
Control EVs carried transcripts that were absent in COVID‐19 groups. (A, B) mRNA transcripts uniquely detected in EVs isolated from Controls but absent in EVs isolated from COVID‐19 groups (grey bars) and differential expression (log 2‐fold change) in other COVID‐19 groups are listed in the heat map (increased expression is red and decreased expression is blue). The general cellular function of each gene product is listed on the right. Large EV transcripts are reported in (A) and small EV transcripts are reported in (B) (*n* = 9–10/group). Independent pairwise comparisons were made between Controls and resolved infection in the first trimester (R1), second trimester (R2), third trimester (R3) or active infection (AI). Transcripts known to be highly abundant in trophoblasts are marked by (T).

There were several interesting transcripts that were only present in large EVs from Controls, including APBA3, MTSS, FCF1, PSG2, LOC100128233, PHOSPHO2 and THOC3 (Figure 6A). These transcripts encode proteins involved in various cellular functions, including signal transduction, transcription and proliferation. In contrast, only a few transcripts were unique to Controls in small EVs. These included PYCR2, SCGB1C1 and CD300L4 (Figure 6B). PYCR2 encodes a cellular metabolism protein; CD300L4 encodes an immune‐regulating protein. The protein function of SCGB1C1 is unknown. Numerous other transcripts were abundant in Controls but absent in individual COVID‐19 groups. If present in the other COVID‐19 groups, their expression was decreased compared to Controls (Figure 6).

Transcripts carried by EVs reflect the activity of the secreting cell. While the individual transcripts identified in EVs differ, their cellular functions often overlap (Table 2 and Figure 6). For example, large and small EVs isolated from R2 carried transcripts that regulate cell signalling, gene expression, immune regulation and metabolism. Additional pathways include proliferation, apoptosis, invasion, ubiquitination, platelet function and vesicle formation.

Many transcripts identified in EVs are abundant in trophoblast cells and have been previously reported to increase with gestation. Interestingly, *THOC3* mRNA, encoded by a highly expressed gene in trophoblast cells, was abundant in Control large EVs but was low or absent in the COVID‐19 groups. In addition, large EVs carried a different highly expressed trophoblast transcript, *RYBP*, in COVID‐19 groups, but this transcript was absent in Controls. Both proteins encoded by these genes are involved in transcriptional regulation (Shu et al. 2022; Wang et al. 2013). Several of the unique mRNAs in EVs isolated from COVID‐19 groups have been previously reported to be associated with adverse pregnancy outcomes, including gestational hypertension, preeclampsia, preterm birth and intra‐uterine growth restriction (Table S6). The number of these pregnancy complication–associated mRNAs was highest in AI EVs, but they were also abundant in EVs isolated from participants with resolved infections. Interestingly, the abundance of eleven transcripts that have been implicated in preeclampsia differed between AI EVs and Control EVs. Of importance, participants with AI also had a higher incidence of preeclampsia. Moreover, two transcripts associated with preterm birth were uniquely carried in R2, but not Control EVs; R2 participants had a higher incidence of preterm birth.

### 3.11 Circulating EVs Carry Transcriptional Regulators of Differentially Expressed Genes in the Placenta

Because EVs had a direct functional effect on trophoblasts in vitro, we investigated whether they carried transcriptional regulators of genes whose expression was altered in the placenta of COVID‐19 pregnant participants (Table S4). Multiple mRNAs encoding transcriptional regulators were differentially abundant in COVID‐19 groups compared to Controls. Several were contained in both small and large EVs (*JUN, FOS, LEPR, LGALS1* and *CD36*) or only in small EVs *(PRKN*). Moreover, Jun proto‐oncogene B (*JUNB*) mRNA levels were increased in large EVs from R1 compared to Controls (FC = 2.25, *p* = 0.01). This suggests that *JUNB* carried in EVs could elicit the observed changes in transcription of its downstream targets in the placenta. Similarly, mRNAs encoding four of the transcriptional regulators in R3 compared to the Control placenta were found in large and small EVs (*IL1B, HIF1A, IGF2* and *PCBP1*); one was only in large EVs (*CXCR4*), and three were only in small EVs (*ESR1, AKT1, TP53, SPZ1* and *IRS2*). Interestingly, interleukin 1 beta (*IL1B*) mRNA was decreased in large EVs from R3 compared to Controls (FC = −1.85, *p* = 0.070), whereas oestrogen receptor 1 (ESR1) mRNA levels were lower in R3 small EVs compared to Controls (FC = −6.99, *p* = 0.13). Importantly, the expression of genes controlled by these transcriptional regulators was altered in the R3 placenta compared to Controls. Similarly, many transcriptional regulators of genes with differential expression in AI compared to Control placenta were found in both large and small EVs (*GRN* and *IL1B*), in large EVs (*FAS*) or in small EVs (*JUN, STAT3, IFG1, IFNG* and *AGT*).

## 4 Discussion

The long‐term effects of COVID‐19 during pregnancy have not yet been elucidated. Previous studies and our findings reported here show that the placenta is damaged, and the likelihood of adverse pregnancy outcomes was increased in patients with a pregnancy complicated by COVID‐19. We have begun to elucidate the mechanisms underlying the observed placental abnormalities associated with COVID‐19. For the first time, we demonstrate that circulating EVs from COVID‐19‐affected pregnancies (1) have a detrimental effect on trophoblast function, including hormone production and invasion in vitro; (2) are altered after SARS‐CoV‐2 infection and (3) carry cargo that has been previously associated with adverse pregnancy outcomes.

Our findings, in in vitro experiments, that trophoblast dysfunction following exposure to EVs isolated from study participants with an active SARS‐CoV‐2 infection provides evidence that circulating EVs contribute to the resulting placental pathology. Previous research has reported that trophoblasts are responsive to EVs (Salomon et al. 2013). We focused our in vitro trophoblast experiments on the response to EVs isolated from participants with an AI because the magnitude of alteration in the placental transcriptome was greater compared to those placentas from resolved infections. Our study did not test the effect of circulating EVs isolated from resolved patients on trophoblast function. EVs from patients with an AI disrupted fundamental trophoblast functions that are crucial to maintain a healthy pregnancy. EVT invasion is vital for anchoring the placenta to the uterus and the remodelling of maternal uterine arteries providing blood to the villous trophoblasts. Inadequate invasion and failure to completely remodel maternal arteries increase the risk of preeclampsia, intra‐uterine growth restriction and foetal loss (Kaufmann et al. 2003; Tantbirojn et al. 2008). Trophoblast hormone production is a key to maintaining a pregnancy. A reduction in the ratio of hCG to progesterone indicates that specific pathways related to steroid hormone production were disrupted. The trophoblast dysfunction caused by AI EVs, including failure to invade and produce hormones, is known to contribute to the development of preeclampsia, preterm birth and intra‐uterine growth restriction (Kaufmann et al. 2003; Tantbirojn et al. 2008). Moreover, a recent publication using spatial transcriptomics reported pathways related to preeclampsia including changes in vascularity are disrupted by COVID‐19 (Stylianou et al. 2024). Therefore, the AI EV–induced reduction in EVT invasion and syncytiotrophoblast hormone production, observed in vitro, may have contributed to the development of these pregnancy complications following COVID‐19. The interpretation of these results is limited because they are based solely on in vitro studies.

Gestational age at the time of infection was a major determinant of COVID‐19‐induced changes in the profile of circulating EVs. If participants were infected during early pregnancy, the number of trophoblast and foetal endothelial cell large EVs were increased and circulating large EVs carried more mtDNA. This suggests that COVID‐19 during early pregnancy disrupts the origin of circulating EVs and EV‐derived cellular mitochondrial function long after the resolution of infection. Our data cannot distinguish between persistently altered EVs in circulation or persistently altered cellular release of EVs and their cargo. However, EV half‐life is thought to be in the order of hours not weeks (Auber and Svenningsen 2022; Frühbeis et al. 2015; Imai et al. 2015; Kang et al. 2021; Rank et al. 2011), thereby suggesting EV release rather than the persistence of EVs in circulation is altered. Mitochondrial dysfunction has been reported in many organs following SARS‐CoV‐2 infection and is thought to contribute to cell injury, cell death and inflammation (Domizio et al. 2022; Faizan et al. 2022; Gibellini et al. 2020). Appelman et al. recently reported persistent mitochondrial dysfunction in skeletal muscle long after the resolution of SARS‐CoV‐2 infection (Appelman et al. 2024). Further, elevated cell‐free circulating mtDNA is commonly observed in COVID‐19 and correlates with severity and length of infection, reflecting significant mitochondria stress (Scozzi et al. 2021; Shoraka et al. 2023; Valdés‐Aguayo et al. 2021; Costa et al. 2022). In support of a direct association of infection and mtDNA release in EVs, Faizan et al. recently demonstrated that SARS‐CoV‐2 infection causes mitochondrial dysfunction and the release of EVs containing mtDNA in airway epithelial cells (Faizan et al. 2022). Thus, our results suggest that abnormal mitochondria may also play a role in the pathogenesis of placental dysfunction in COVID‐19.

Circulating EV cargo reflects cellular activity. The transcripts carried by EVs encode genes related to inflammation, vasculopathies, bioenergetics and cell death, processes and pathways that were present in the transcriptome and histopathology of the placenta regardless of the timing of infection. Within the total EV subpopulations, there are transcripts that are highly expressed by trophoblasts and have known functions in cellular metabolism, immune regulation and transcription. We also found that many of the transcripts in EVs from pregnancies complicated by COVID‐19 are encoded by genes that have been implicated in adverse pregnancy outcomes including gestational hypertension, preeclampsia, preterm birth and intra‐uterine growth restriction. This points to shared pathways of placental dysfunction induced by a systemic SARS‐CoV‐2 infection.

EV cargo can elicit a functional response when delivered to a recipient cell, as demonstrated by our in vitro studies. While it is not known if mtDNA in EVs per se was responsible for altering trophoblast function in our experiments, multiple studies have demonstrated that mitochondria cargo can alter the recipient cell's mitochondrial function (Peruzzotti‐Jametti et al. 2021; Phinney et al. 2015; Sansone et al. 2017). mRNA transcripts are also biologically active in recipient cells, and we identified transcripts in EVs that encode for multiple transcriptional regulator genes whose expression was altered in the placenta following COVID‐19. Importantly, the expression of several of these genes has been previously reported to be altered in pregnancy complications. For example, JUN signalling was disrupted in the R1 placenta compared to the Control placenta, and JUNB mRNA was increased in Control compared to R1 large EVs. JUN proteins are important for placentation, and a loss of JUN signalling is implicated in preeclampsia (Schorpp‐Kistner et al. 1999; Nuzzo et al. 2014). Thus, low levels of JUNB in COVID‐19 EVs may indicate placental dysfunction, which in turn could contribute to the later development of preeclampsia, which is observed at higher rates in pregnancies complicated by COVID‐19 (Papageorghiou et al. 2021). In R3 compared to Controls, hormone receptor signalling was identified as a top canonical pathway, and differentially expressed genes were regulated by ESR1. *ESR1* mRNA was abundant in small EVs isolated from Controls but not R3. ESR1 signalling is vital for placental function and pregnancy maintenance because oestrogen signalling is obligate for angiogenesis and vasculature control (Berkane et al. 2017). In fact, genetic variations in *ESR1* are associated with recurrent pregnancy loss and preeclampsia, and both adverse pregnancy outcomes are increased in maternal SARS‐CoV‐2 infection during pregnancy (Bahia et al. 2020; El‐Beshbishy et al. 2015). These observations exemplify potential EV‐driven signalling leading to altered gene expression in the placenta that occurs in COVID‐19.

The effects of COVID‐19 are known to be long‐lasting in many tissues. Here, we report that COVID‐19‐induced placenta pathology is evident even months after infection. Interestingly, high‐grade MVM and perivillous fibrin deposition were increased in the placenta of subjects who recovered from a SARS‐CoV‐2 infection during the second or third trimester. This is an example of a difference in the effect of SARS‐CoV‐2 infection earlier in the third trimester (R3) compared to later in the third trimester (AI). This suggests that these lesions may take weeks to manifest. The placenta collected from subjects who experienced an infection in the first trimester do not have evidence of these lesions, suggesting either resilience or eventual attenuation. Transcriptional changes in the placenta are also evident long after COVID‐19. Many disrupted pathways are common in the placentas regardless of the timing of infection. The results of our experiments cannot distinguish between persistent damage and ongoing pathology. However, we also found long‐lasting effects on circulating EVs suggesting the potential for persistent EV‐mediated pathology.

Our study is limited by the number of symptomatic patients with an AI at the time of delivery, thereby limiting our ability to assess the effect of severity on placental dysfunction and EV characteristics. Despite only 14% of pregnant participants experiencing COVID‐19‐related symptoms and all having mild cases as defined by the NIH criteria, their placentas had significant pathology and an altered transcriptome. This was associated with an increased incidence of preeclampsia and medically indicated preterm birth in asymptomatic and symptomatic AI cases. It is worth noting that EVs obtained from asymptomatic individuals have been found to exert significant impacts on trophoblast function when tested in vitro. This discovery highlights the importance of exploring the potential consequences of EV exposure in asymptomatic patients and may have important implications for understanding the role of EVs in reproductive health.

The number of subjects who experienced an adverse pregnancy outcome is limited in our study. This limits our interpretation of the risk of pregnancy complications following COVID‐19 at specific trimesters. The small number of subjects also limits our interpretation of the role of specific EV‐associated transcripts that are also previously associated with pregnancy complications. It is striking to observe many transcripts associated with pregnancy complications, particularly IUGR, but a relatively low rate of IUGR. The complexity of IUGR pathogenesis may explain why so few pregnancies are diagnosed with IUGR but have potential circulating factors associated with IUGR. We are also underpowered to test for the effect of other factors that are known to impact the risk for placental dysfunction and pregnancy complications such as maternal race and BMI, parity and infant sex. Future studies are warranted to evaluate the influence of these factors on COVID‐19‐induced placental dysfunction and altered circulating EV characteristics.

Our study has provided significant insights into the profile and functional consequences of circulating EVs in mothers who were infected with SARS‐CoV‐2. This study is the first to demonstrate the negative impact of maternal circulating vesicles on trophoblast function in COVID‐19. By comparing the placental transcriptome and EV cargo content, we have identified shared pathways that are associated with pregnancy complications caused by maternal COVID‐19 and other pregnancy‐related disorders that are not well understood.

## Author Contributions

**Thea N. Golden**: Conceptualization (Equal), Data curation (Lead), Formal analysis (Lead), Funding acquisition (Equal), Investigation (Lead), Methodology (Lead), Project administration (Equal), Supervision (Equal), Validation (Equal), Visualization (Lead), Writing – original draft (Lead), Writing – review and editing (Equal). **Sneha Mani**: Conceptualization (supporting), Formal analysis (supporting), investigation (supporting), methodology (supporting), validation (supporting), writing – review and editing (supporting). **Rebecca L. Linn**: Conceptualization (supporting), Formal analysis (Supporting), Investigation (Supporting), Methodology (Supporting), Resources (Supporting), Validation (Supporting), Writing – review and editing (Supporting). **Rita Leite**: Investigation (supporting), resources (supporting), writing – review and editing (supporting). **Natalie A. Trigg**: Software (Supporting), Writing – review and editing (Supporting). **Annette Wilson**: Investigation (supporting), writing – review and editing (supporting). **Lauren Anton**: Investigation (supporting), methodology (supporting), writing – review and editing (supporting). **Monica Mainigi**: Conceptualization (supporting), resources (supporting), writing – review and editing (supporting). **Colin C. Conine**: Data curation (Supporting), Formal analysis (Supporting), Methodology (Supporting), Resources (Supporting), Software (Lead), Writing – review and editing (Supporting). **Brett A. Kaufman**: Conceptualization (Supporting), Formal analysis (Supporting), Funding acquisition (Supporting), Methodology (Supporting), Resources (Supporting), Validation (Supporting), Writing – review and editing (Supporting). **Jerome F. Strauss III**: Conceptualization (Supporting), Resources (Supporting), Supervision (Supporting), Writing – review and editing (Supporting). **Samuel Parry**: Conceptualization (supporting), funding acquisition (equal), project administration (equal), resources (supporting), supervision (equal), writing – review and editing (supporting). **Rebecca A. Simmons**: Conceptualization (Equal), Funding acquisition (Equal), Project administration (Equal), Supervision (Equal), Validation (Equal), Writing – original draft (Supporting), Writing – review and editing (Equal).

## Conflicts of Interest

The authors declare no conflicts of interest.

## Supporting information



Supplemental Table 1. Pregnancy Outcomes
Supplemental Table 2. Placenta RNAseq DEGs
Supplemental Table 3. Placenta RNAseq canonical pathways
Supplemental Table 4. Placental RNAseq transcriptional regulators
Supplemental Table 5. BeWo RNAseq pathway analysis
Supplemental Table 6. EV Transcripts Associated with Adverse Pregnancy Outcomes

## Data Availability

The sequencing data reported in this study are deposited in GEO (GSE268230). All other data are available in the main text.
